# Effects of airway pressure release ventilation on multi-organ injuries in severe acute respiratory distress syndrome pig models

**DOI:** 10.1186/s12890-022-02238-x

**Published:** 2022-12-07

**Authors:** Aijia Ma, Bo Wang, Jiangli Cheng, Meiling Dong, Yang Li, Canzheng Wei, Yongfang Zhou, Yang Xue, Hui Gao, Lican Zhao, Siyu Li, Yiwei Qin, Mengni Zhang, Qin Wu, Jing Yang, Yan Kang

**Affiliations:** 1 Department of Critical Care Medicine, West China Hospital of Sichuan University, No. 37, Guoxue Alley, Chengdu, 610041 Sichuan Province China; 2 Department of Nursing, West China Hospital of Sichuan University, Chengdu, Sichuan Province China; 3 Department of Critical Care Medicine, The First Affiliated Hospital of Chengdu Medical College, Chengdu, Sichuan Province China; 4 Department of Pathology, West China Hospital of Sichuan University, Chengdu, Sichuan Province China

**Keywords:** Acute respiratory distress syndrome, Mechanical ventilation, Airway pressure release ventilation, Low tidal volume, Multi-organ dysfunction syndrome

## Abstract

**Background:**

Extra-pulmonary multi-organ failure in patients with severe acute respiratory distress syndrome (ARDS) is a major cause of high mortality. Our purpose is to assess whether airway pressure release ventilation (APRV) causes more multi-organ damage than low tidal volume ventilation (LTV).

**Methods:**

Twenty one pigs were randomized into control group (n = 3), ARDS group (n = 3), LTV group (n = 8) and APRV group (n = 7). Severe ARDS model was induced by repeated bronchial saline lavages. Pigs were ventilated and monitored continuously for 48 h. Respiratory data, hemodynamic data, serum inflammatory cytokines were collected throughout the study. Histological injury and apoptosis were assessed by two pathologists.

**Results:**

After severe ARDS modeling, pigs in ARDS, LTV and APRV groups experienced significant hypoxemia and reduced lung static compliance (C_stat_). Oxygenation recovered progressively after 16 h mechanical ventilation (MV) in LTV and APRV group. The results of the repeated measures ANOVA showed no statistical difference in the PaO_2_/FiO_2_ ratio between the APRV and LTV groups (*p* = 0.54). The C_stat_ showed a considerable improvement in APRV group with statistical significance (*p* < 0.01), which was significantly higher than in the LTV group since 16 h (*p* = 0.04). Histological injury scores showed a significantly lower injury score in the middle and lower lobes of the right lung in the APRV group compared to LTV (*p*_middle_ = 0.04, *p*_lower_ = 0.01), and no significant increase in injury scores for extra-pulmonary organs, including kidney (*p* = 0.10), small intestine (*p* = 1.0), liver (*p* = 0.14, *p* = 0.13) and heart (*p* = 0.20). There were no significant differences in serum inflammatory cytokines between the two groups.

**Conclusion:**

In conclusion, in the experimental pig models of severe ARDS induced by repetitive saline lavage, APRV improved lung compliance with reduced lung injury of middle and lower lobes, and did not demonstrate more extra-pulmonary organ injuries as compared with LTV.

**Supplementary Information:**

The online version contains supplementary material available at 10.1186/s12890-022-02238-x.

## Background

Acute respiratory distress syndrome (ARDS) is a severe life-threatening respiratory disorder in the intensive care unit (ICU), associated with a high mortality and a massive economic burden [1, 2]. Recent studies have reported a 46–60% mortality rate for severe ARDS patients in ICU [3]. However, the predominant cause of death in severe ARDS does not appear to be severe hypoxemia, but rather the multi-organ dysfunction syndrome (MODS) [4, 5]. Several studies have confirmed that sepsis and MODS are the most common causes of death in severe ARDS (30–50%), while irreversible respiratory failure accounted for only 13–19% [6]. The kidneys (40–55%), liver (12–95%), brain (12–95%), intestines (7–30%), and heart (40–55%) are among the organs that may fail secondary to the course of severe ARDS [7].

Decades of research failed to find effective therapies that reduced mortality in ARDS, and the most crucial supportive measure is still mechanical ventilation (MV). However, It has been proposed that MV may contribute to the onset of MODS by affecting hemodynamics [8, 9], releasing inflammatory mediators from the lung into the bloodstream [10, 11], increasing alveolar-vascular permeability [12], and causing endotoxin or bacterial translocation [13, 14]. Therefore, the use of protective ventilation strategies is crucial. And there is an urgent need to explore whether there is an ideal ventilation mode that can alleviate lung conditions while minimizing damage to other extra-pulmonary organs, thereby reducing the occurrence of MODS.

Low tidal volume ventilation (LTV) is current a generally recognized protective ventilation strategy, using a lower tidal volume to prevent excessive lung inflation [15]. In 2017, it was strongly recommended by the American Thoracic Association as the preferred treatment for ARDS [16]. Although adequate lung protection is given, there is no trend to reduce MODS, the mortality of severe ARDS still remains high. Airway pressure release ventilation (APRV) is a mode of ventilation that involves maintaining a continuous high positive pressure for most of the cycle with intermittent release phases, while allowing for spontaneous respiration [17]. APRV has been recognized as an effective ventilation strategy in recent years, which even shows better oxygenation benefits than conventional LTV. Our team’s previous studies have shown that early application of APRV in patients with ARDS can reduce the duration of mechanical ventilation and improve pulmonary permeability [18, 19].

Despite the benefits of “open the lung” strategy in APRV, there is concern that exposure to high trans-pulmonary pressures, may raise the risk of hemodynamic instability, which in turn cause damage to extra-pulmonary organs and drive the development of MODS [20]. But the evidence on whether APRV causes extra-pulmonary organ damage is scarce. There appears to be a greater emphasis on pulmonary protection while ignoring if APRV has deleterious effects on extrapulmonary organs, which is crucial to death from MODS.

Therefore, we evaluated the histological changes of lung, kidney, small intestine, liver, and heart samples obtained from severe ARDS pig models, which treated with two modes of mechanical ventilation (LTV and APRV) for 48 h.

The aim of this study was to determine whether the still controversial APRV causes more extra-pulmonary multi-organ damage than conventional LTV due to its sustained inspiratory high pressure, hence raising the possibility of MODS.

## Methods

**Fig. 1:**
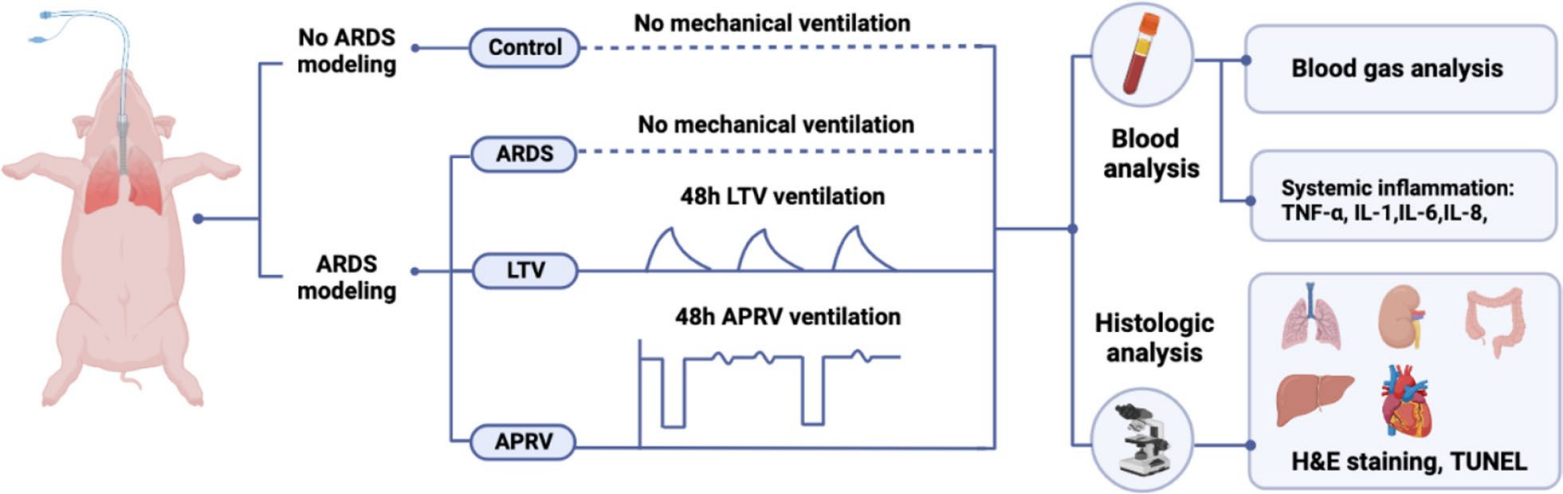
Summary of the experimental design of this study

All experiments and methods had been reviewed and approved by the animal experiment ethics committee of Sichuan University (2018073A). Animals were sourced from the Laboratory Animal Center of Sichuan University, China. The care and execution of the research animals complied with the ARRIVE guideline for laboratory animals [21]. The summary of this study is shown in Fig. 1.

### Animal preparation

Female Bama mini swine, weighing 30–35 kg, were fasted for 12 h but allowed free access to water before the experiments. After premedication with intramuscular atropine (0.02–0.05 mg/kg), general anesthesia was administered by ear vein injection of sufentanil (0.2 μg/kg/h), midazolam (0.2 mg/kg/h) and propofol (0.3–1 mg/kg/h). Sedation was aimed at no agitation, no respiratory distress, and no hypotension or bradycardia due to deep analgesia.

A continuous intravenous infusion of balanced electrolyte solution or saline (2–4 ml/kg/h) was administered to maintain daily physiological requirements and norepinephrine (0.02–1 μg/kg/min) was titrated to maintain a mean arterial pressure (MAP) > 80 mmHg in the pigs for the duration of the experiment. Tracheotomy was performed using a 7.0 mm inner diameter tracheotomy catheter connected to a ventilator in all animals (Puritan Bennett™ 840, Medtronic, USA) for baseline settings.

All animals were initially ventilated with baseline setting: volume assist-controlled ventilation mode (A/C-VCV), tide volume (V_T_), 6–8 mL/kg; ventilator setting frequency, 12–16 cycle/min; positive end-expiratory pressure (PEEP), 5 cm H_2_O; fraction of inspired oxygen (FiO_2_), 40%.

### Study groups

Following baseline stabilization, the animals were randomly allocated into one of four groups:

1. Control group (n = 3): Only performed animal preparation, no severe ARDS modeling and mechanical ventilation. The control group was set up to observe normal organs at the histological level and to eliminate the interference of ARDS disease and 48 h mechanical ventilation.
2. ARDS group (n = 3): Performed animal preparation and severe ARDS modeling without 48 h mechanical ventilation. The ARDS group was set up to observe organ damage from the ARDS disease at a histological level, which eliminated the intervention of mechanical ventilation on the disease.
3. LTV group (n = 8): Severe ARDS modeling with 48 h mechanical ventilation using LTV ventilation mode.
4. APRV group (n = 7): Severe ARDS modeling with 48 h mechanical ventilation using APRV ventilation mode.

### ARDS induction

A severe ARDS model was induced in ARDS, LTV, and APRV groups by repetitive intratracheal installation of warmed (37.5 °C) normal saline to produce lung injury, which was saline lavage method [22]. The fiberoptic bronchoscope infuses saline into the lungs through the tracheotomy catheter. Each lung segment was about 10–50 ml of 90% saline (60 ml/kg saline in total). After the injection, the lavage fluid was quickly sucked out. The recovery rate of alveolar lavage fluid should reach 50–60% of the total fluid. If the blood gas analysis was stably maintained at PaO_2_/FiO_2_ < 100 mmHg for 30 min, the establishment of severe ARDS model was considered to be completed.

### Ventilator setting in LTV group

The initial setting of LTV group was basically consistent with the baseline setting of A/C-VCV. The PEEP and oxygen concentration were adjusted sequentially according to the Higher PEEP/Lower FiO_2_ table recommended by ARDSnet. Then, PEEP could be further titrated by the ways of optimum respiratory compliance (C_stat_). The ventilator setting frequency and V_T_ were titrated to achieve the goal of PaO_2_, 55–100 mmHg, PaCO_2_, 30–50 mmHg, arterial pH ≥ 7.30, and the plateau airway pressure (P_plat_) ≤ 30 cm H_2_O, according to the ARDSnet protocol. If PaO_2_/FiO_2_ ratio was less than 150 mmHg, recruitment maneuver would be considered. If the animals presented severe respiratory acidosis (pH < 7.15), the respiratory rate could be increased to 35 cycle/min. A more detailed LTV parameter adjustment strategy has been described in the Additional file 1.

### Ventilator setting in APRV group

High airway pressure (P_high_) set to P_plat_ measured at the baseline setting, not to exceed 30 cm H_2_O. Low airway pressure (P_low_) set to 5 cm H_2_O. The duration of the release phase (T_low_) initially set at 1–1.5 times the expiratory time constant and then adjusted to achieve ≥ 50% of the terminated peak expiratory flow rate (PEFR). The release frequency was 10–14 frequency/min; The duration of P_high_ (T_high_) was calculated indirectly from T_low_ and release frequency. If the pig-ventilator asynchrony appeared and V_T_ is less than 6 ml/kg, it is permitted to gradually extend T_low_ in order to maintain a termination of expiratory flow rate. The target for the level of spontaneous breathing was approximately 30% of the total minute ventilation.

The 48 h parameters of mechanical ventilation in APRV group were not static but fine-tuned according to respiratory mechanics, ventilation parameters, arterial blood gas analysis, hemodynamic parameters. For full experimental details on the ventilator settings for titration, see Additional file 1.

### Data collection

Vital signs and hemodynamic data were recorded every hour, arterial blood gas analysis and pulmonary mechanics were measured every 4 h. The blood sample were obtained at baseline (T0), severe ARDS modeling (T1), 24 h and 48 h of the study period (T2, T3, respectively).

### Blood sample analysis

Whole blood samples (20 µl) were analyzed by hemodilution with diluent and then measured with a MEK-6318 K automated hematocrit analyzer (Nihon Kohden Corp.). Tumor necrosis factor alpha (TNF-α), interleukin 1 (IL-1) interleukin 6 (IL-6) and interleukin 8 (IL-8) in serum were detected by ELISA kits from Shanghai Zoucai Biotechnology Co.

### Tissue sample collection and preparation

The animals were sacrificed by perfusion with frozen normal saline containing heparin sodium (12,500 µ/L). Tissue samples were collected for subsequent experiments.

Lung tissues from the right lung segment (upper, middle, lower), kidney tissue at the junction of the left renal pelvis and calyces, small intestine tissues from the ileum, liver tissues and left ventricular tissue were randomly selected at 10 sites and placed in lyophilization tubes, subsequently stored in a -80℃ refrigerator.

### Histopathological analysis

The tissues were sectioned at a thickness of 4 µm, stained with hematoxylin and eosin (H&E). Histological images were taken under a microscope with 200 × and 400 × fields of view (Axio Scope, Zeiss, Germany). Different organs were analyzed quantitatively according to different scoring criteria [23–27], as displayed in Table 1. For histological analysis, 5 slides were randomly selected, and 5 visual fields were imaged per slide to minimize bias. The analysis of the results was assessed by two pathologists separately and averaged. A senior pathologist will be invited to judge when the two disagree.

**Table 1:** Criteria for the Microscopic Scoring of Tissue Damage

Organ	Method	Parameters
Lung	American Thoracic Society's published lung tissue semi-quantitative injury score [23]	5 parameters: A, neutrophils in the alveolar lumen; B, neutrophils in the interstitium; C, hyaline membrane changes; D, protein debris in the alveolar lumen; E, alveolar wall thickening Score = [(20 × A) + (14 × B) + (7 × C) + (7 × D) + (2 × E)]/number of visual fields
Kidney	Paller score [24]	6 parameters: A, large dilated tubules with flattened cells = 1; B, tubular pattern = 2; C, necrotic, detached cells in tubular lumen = 1; D, granular degeneration of epithelial cells = 1; E, vacuolar degeneration = 1; F, nuclear consolidation = 1
Intestine	Chiu score [25]	5 points, according to the separation of mucosal epithelium and lamina propria, inflammatory cells, bleeding degree
Liver	Hepatic shock score and fat infiltration score [26]	Hepatic shock score, 4 points, A, normal = 1; B, central lobule congestion without necrosis = 2; C, central lobule congestion and central lobule necrosis = 3; D, three or more adjacent lobules necrotic = 4 Fatty infiltration score, 4 points, A, no vacuoles in hepatocytes = 1; B, less than 50% of hepatocytes have single or several vacuoles = 2; C, less than 50% of hepatocytes have multiple vacuoles = 3; D, more than 50% of hepatocytes have multiple vacuoles in them =
Heart	Heart injury score [27]	5 parameters, according to edema, degeneration, inflammation, congestion, and subendocardial hemorrhage

### Apoptosis analysis

Organ paraffin slices were used to quantify apoptosis using terminal deoxynucleotidyl transferase dUTP nick end labeling (TUNEL). Propidium iodide (PI) was used to stain the nuclei, and TUNEL was used to identify cells that had undergone apoptosis. Five fields from each section were randomly chosen for counting. The apoptotic index of five organs were calculated as [100% both TUNEL and PI-positive apoptotic nuclei)/ (PI-positive nuclei)] [28].

### Statistical analysis

The SPSS Statistics Software 25.0 was used to process all quantitative experimental data. Respiratory and hemodynamic parameters were described using mean and standard deviation.

Differences in respiratory and hemodynamic variables at T0, T1, T2 and T3 were compared between groups using the Kruskal–Wallis H-test. The continuous variables of 48 h were calculated by repeated measures ANOVA. Post hoc Bonferroni tests were performed at specific time points only if significance was found in the group*time effect using repeated measures ANOVA. Differences between groups were detected using Kruskal–Wallis H-test for injury scores and apoptotic index, and the results were adjusted for significance values by the Bonferroni method, the adjusted *p* < 0.05 were considered significant.

## Results

### Effect of mechanical ventilation on respiratory and hemodynamic variables

**Fig. 2:**
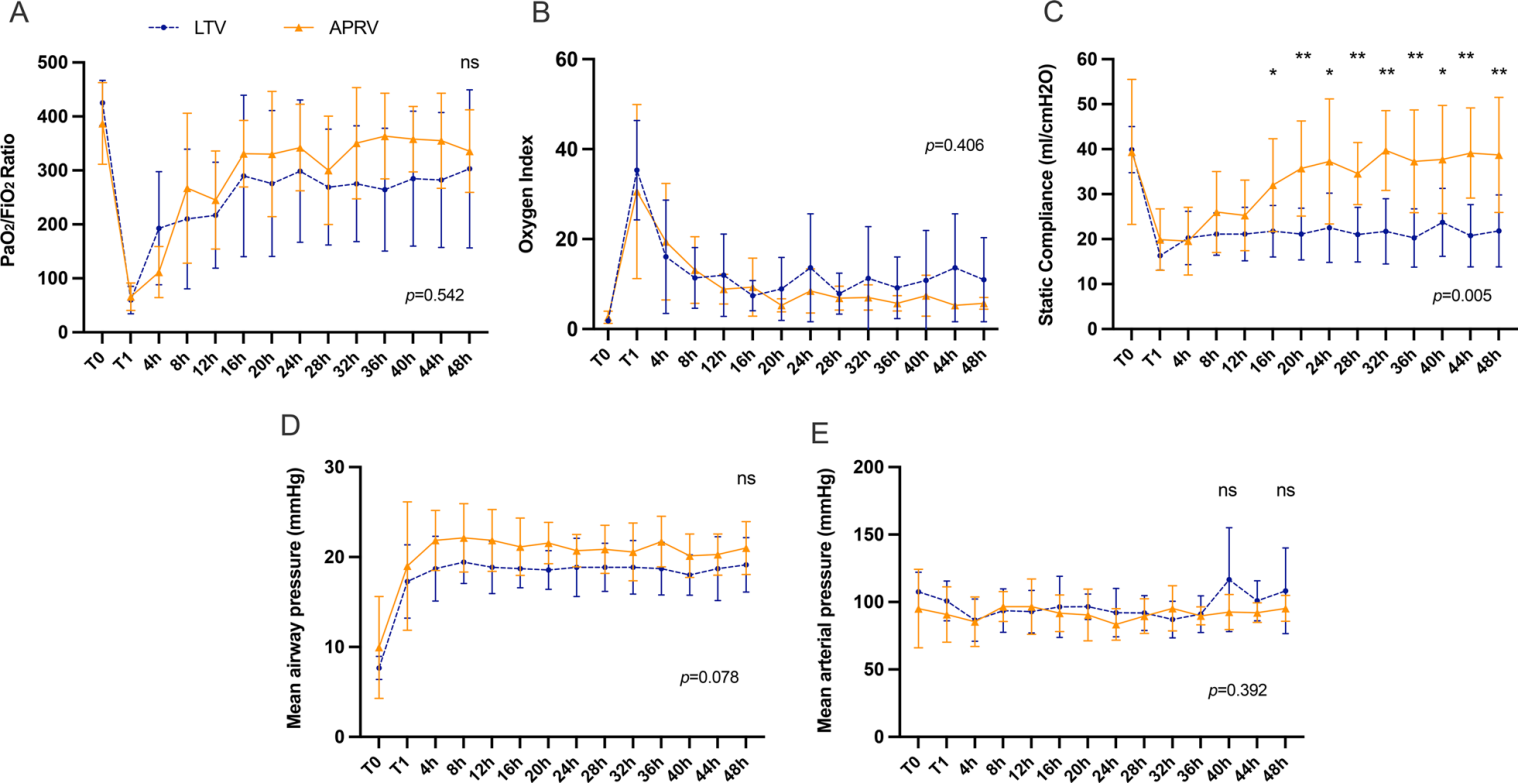
Respiratory and hemodynamic variables at different time points. **A** PaO_2_/FiO_2_ ratio; **B** Oxygen index; **C** Static compliance of the respiratory system; **D** Mean airway pressure; **E** Mean arterial pressure; The *p*-values were calculated by repeated measurement ANOVA between the two groups; *p < 0.05 compared to the LTV group at diffeerent time point in Bonferroni post-hoc test; ns = no significance

Table 2 demonstrates all data of respiratory variables. ARDS, LTV and APRV groups experienced significant hypoxemia and reduced compliance followed the modeling of ARDS (T1). Oxygenation recovered progressively after 16 h in LTV and APRV groups. The results of the repeated measures ANOVA showed no statistical difference in the PaO_2_/FiO_2_ ratio between the APRV and LTV groups (*p* = 0.54) (Table 2, Fig. 2A). The oxygen index, calculated as P_mean_*FiO_2_/PaO_2_, is considered to be a more comprehensive method of assessing oxygenation.The repeated measures ANOVA results showed that there was no statistical difference in oxygen index between the APRV and LTV groups during 48 h of mechanical ventilation (*p* = 0.41) (Fig. 2B).

**Table 2:** Respiratory variables

Group	Time			
	T0	T1	T2	T3
*Ventilator setting V*_*T*_* (ml)*				
CON	320.0 (70.0)	/	/	/
ARDS	283.2 (15.3)	266.7 (41.6)	/	/
LTV	272.9 (52.8)	233.3 (30.6)	250.0 (26.2)	233.8 (24.5)
APRV	267.1 (45.7)	/	/	/
*Ventilator setting frequency (cycles/min)*				
CON	15.3 (4.2)	/	/	/
ARDS	16.0 (0.0)	19.0 (6.6)	/	/
LTV	20.6 (5.4)	33.7 (5.5)	29.6 (5.0)	25.9 (6.6)
APRV	22.4 (5.3)	21.7 (0.6)*	19.7 (4.8)*	19.9 (4.5)*
*Total minute ventilation (L/min)*				
CON	5.0 (0.5)	/	/	/
ARDS	4.5 (0.3)	8.3 (3.2)	/	/
LTV	5.3 (1.9)	10.4 (3.7)	7.2 (1.9)	7.2 (1.8)
APRV	6.2 (1.9)	7.1 (3.7)	6.0 (3.0)	7.8 (3.9)
*P*_*high*_* (cmH*_*2*_*O)*				
APRV	/	24.7 (3.5)	24.6 (1.7)	23.9 (2.0)
*PEEP (mmHg)*				
CON	5.0 (0.0)	/	/	/
ARDS	5.0 (0.0)	5.3 (0.6)	/	/
LTV	5.0 (0.0)	13.7 (1.5)	13.9 (1.7)	13.9 (1.9)
APRV	5.0 (0.0)	5.0 (0.0)*	5.0 (0.0)*	5.3 (0.8)*
*T*_*low*_* (s)*				
APRV	/	0.4 (0.0)	0.4 (0.1)	0.4 (0.1)
*Setting FiO*_*2*_* (%)*				
CON	36.7 (5.8)	/	/	/
ARDS	38.4 (10.4)	43.7 (50.9)	/	/
LTV	37.1 (11.1)	100.0 (0.0)	14.1 (19.2)	47.5 (27.0)
APRV	36.7 (5.8)	100.0 (0.0)	23.1 (29.0)	35.0 (7.6)
*Respiratory rate (breaths/min)*				
CON	15.3 (4.2)	/	/	/
ARDS	16.0 (0.0)	22.3 (11.7)	/	/
LTV	21.3 (5.5)	38.0 (3.5)	29.9 (4.7)	28.9 (6.7)
APRV	22.4 (5.3)	28.0 (8.9)	20.5 (4.7)*	20.9 (3.7)*
*Peak inspiratory pressure (cmH*_*2*_*O)*				
CON	16.7 (0.6)	/	/	/
ARDS	14.0 (2.6)	27.0 (14.7)	/	/
LTV	14.8 (3.7)	32.3 (5.9)	30.5 (6.7)	29.3 (8.3)
APRV	21.3 (9.5)	24.7 (3.5)	26.6 (2.6)	25.6 (3.2)
*Mean airway pressure (cmH*_*2*_*O)*				
CON	8.2 (0.7)	/	/	/
ARDS	7.3 (0.2)	12.3 (6.0)	/	/
LTV	7.7 (1.3)	20.3 (2.5)	19.3 (3.2)	19.5 (2.9)
APRV	12.3 (6.2)	21.3 (3.5)^§^	21.4 (2.5)	21.3 (3.0)
*Static compliance (ml/cmH*_*2*_*O)*				
CON	32.7 (5.1)	/	/	/
ARDS	42.3 (10.1)	25.3 (20.5)	/	/
LTV	39.9 (5.1)	14.3 (2.1)	22.5 (7.7)	21.8 (8.0)
APRV	39.4 (16.1)	24.3 (9.0)	37.3 (13.9)*	38.7 (12.8)*
*pH*				
CON	7.4 (0.1)	/	/	/
ARDS	7.5 (0.1)	7.3 (0.1)	/	/
LTV	7.4 (0.1)	7.4 (0.1)	7.4 (0.1)	7.4 (0.1)
APRV	7.4 (0.1)	7.4 (0.1)	7.5 (0.1)	7.5 (0.1)
*PaCO*_*2*_* (mmHg)*				
CON	40.1 (8.0)	/	/	/
ARDS	37.1 (11.7)	44.1 (9.2)	/	/
LTV	48.7 (15.0)	48.1 (14.5)	52.4 (14.1)	49.0 (11.7)
APRV	46.7 (15.3)	49.2 (8.9)	45.6 (13.6)	40.6 (7.0)
*PaO*_*2*_*/FiO*_*2*_* Ratio*				
CON	415.0 (41.1)	/	/	/
ARDS	445.7 (19.7)	46.5 (2.1)	/	/
LTV	433.3 (51.0)	59.9 (19.6)	298.7 (132.0)	303.0 (146.5)
APRV	349.3 (115.3)	65.9 (25.4)	342.4 (80.3)	335.7 (76.4)

Data are presented as the mean(standard deviation) of the values recorded at T0 (before severe ARDS modeling), T1 (after severe ARDS modeling), T2 (24 h mechanical ventilation after severe ARDS modeling) and T3(48 h mechanical ventilation after severe ARDS modeling); CON, control group; ARDS, severe acute respiratory distress syndrome group; LTV, low tidal volume ventilation group; APRV, airway pressure release ventilation group; V_T_, tidal volume; P_high_, pressure during inspiration; T_low_, the time spent at/release phase; FiO_2_, fraction of inspired oxygen; Total minute ventilation including release minute ventilation + spontaneous minute ventilation; ^§^*p* < 0.05 compared to the ARDS group at this time point in Kruskal–Wallis H-test; *p < 0.05 compared to the LTV group at this time point in Kruskal–Wallis H-test

The C_stat_ showed a considerable improvement in APRV group with statistical significance (*p* < 0.01), which was significantly higher than in the LTV group since 16 h (*p* = 0.04) (Table 2, Fig. 2C).

The result of P_mean_ suggested the difference between the APRV and LTV groups was not statistically significant (*p* = 0.08) (Fig. 2D). P_peak_ was not higher than the LTV group because of the ventilator setting of the APRV group, and both groups maintained similar total minute ventilation at all time points (Table 2).

Hemodynamic data including heart rate, cumulative fluids and urine volume are shown in Table 3, which the results showed no difference between the two groups. The mean arterial pressure in APRV group was not significantly different from LTV group during 48 h ventilation (*p* = 0.49) (Fig. 2E).

**Table 3:** Hemodynamic variables

Group	Time			
	T0	T1	T2	T3
*Heart rate (beats/min)*				
CON	89.0 (16.5)	/	/	/
ARDS	90.0 (18.2)	132.0 (8.5)	/	/
LTV	73.0 (23.0)	135.3 (11.8)	93.9 (37.3)	91.1 (31.5)
APRV	67.3 (12.3)	154.7 (46.5)	94.1 (26.2)	84.3 (13.3)
*Systolic blood pressure (mmHg)*				
CON	132.0 (33.3)	/	/	/
ARDS	137.0 (25.4)	139.0 (8.5)	/	/
LTV	140.4 (17.4)	148.0 (7.5)	129.5 (14.9)	144.5 (26.1)
APRV	128.9 (29.8)	133.3 (28.9)	120.3 (12.4)	127.4 (12.6)
*Diastolic blood pressure (mmHg)*				
CON	73.3 (32.1)	/	/	/
ARDS	80.0 (14.4)	92.5 (4.9)	/	/
LTV	84.0 (11.9)	88.3 (16.2)	129.5 (14.9)	83.6 (33.2)
APRV	70.7 (25.5)	82.3 (23.1)	60.1 (12.4)	84.4 (28.4)
*Mean arterial pressure (mmHg)*				
CON	98.0 (43.3)	/	/	/
ARDS	105.0 (18.0)	110.0 (4.2)	/	/
LTV	107.6 (14.5)	109.0 (14.4)	92.1 (17.9)	108.3 (31.8)
APRV	95.1 (29.1)	103.7 (25.7)	83.4 (11.7)	95.3 (9.6)
*Cumulative fluids (L)*				
LTV	/	2.6 (2.4)	1.9 (1.1)	2.9 (1.4)
APRV	/	2.1 (1.5)	1.8 (0.7)	3.2 (1.8)
*Unrine volume (mL)*				
LTV	/	77.9 (26.1)	58.7 (33.5)	63.1 (59.0)
APRV	/	70.7 (52.1)	100.1 (69.4)	81.8 (41.8)

Data are presented as the mean(standard deviation) of the values recorded at T0 (before severe ARDS modeling), T1 (after severe ARDS modeling), T2 (24 h mechanical ventilation after severe ARDS modeling) and T3(48 h mechanical ventilation after severe ARDS modeling); CON, control group; ARDS, severe acute respiratory distress syndrome group; LTV, low tidal volume ventilation group; APRV, airway pressure release ventilation group; *p < 0.05 compared to the LTV group at this time point in Kruskal–Wallis H-test

### Histopathological analysis in multi-organ tissues due to mechanical ventilation in ARDS

#### Lung

**Fig. 3:**
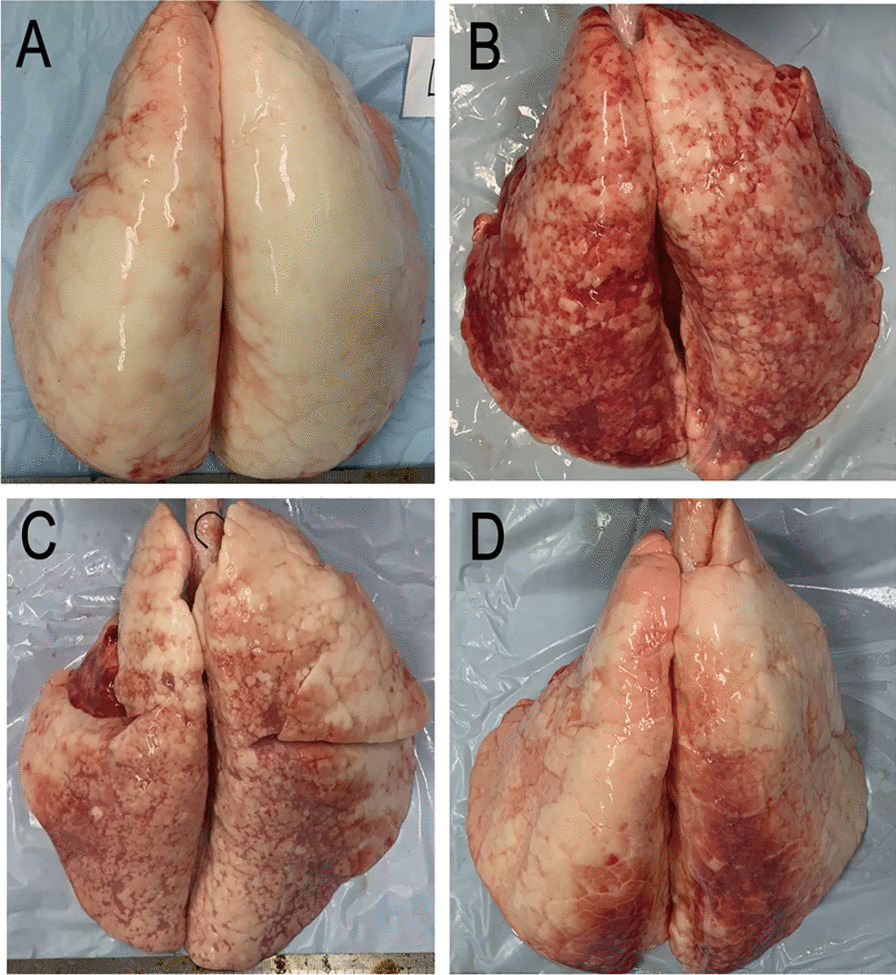
Lung tissue samples in different groups. **A** Control group; **B** ARDS group; **C** LTV group; **D** APRV group

The typical pathological changes of lung tissues in four groups are shown in Fig. 3. The lung consolidation showed a typical severe ARDS gravity distribution, the lower lung consolidation in ARDS, LTV and APRV group were severe.

**Fig. 4:**
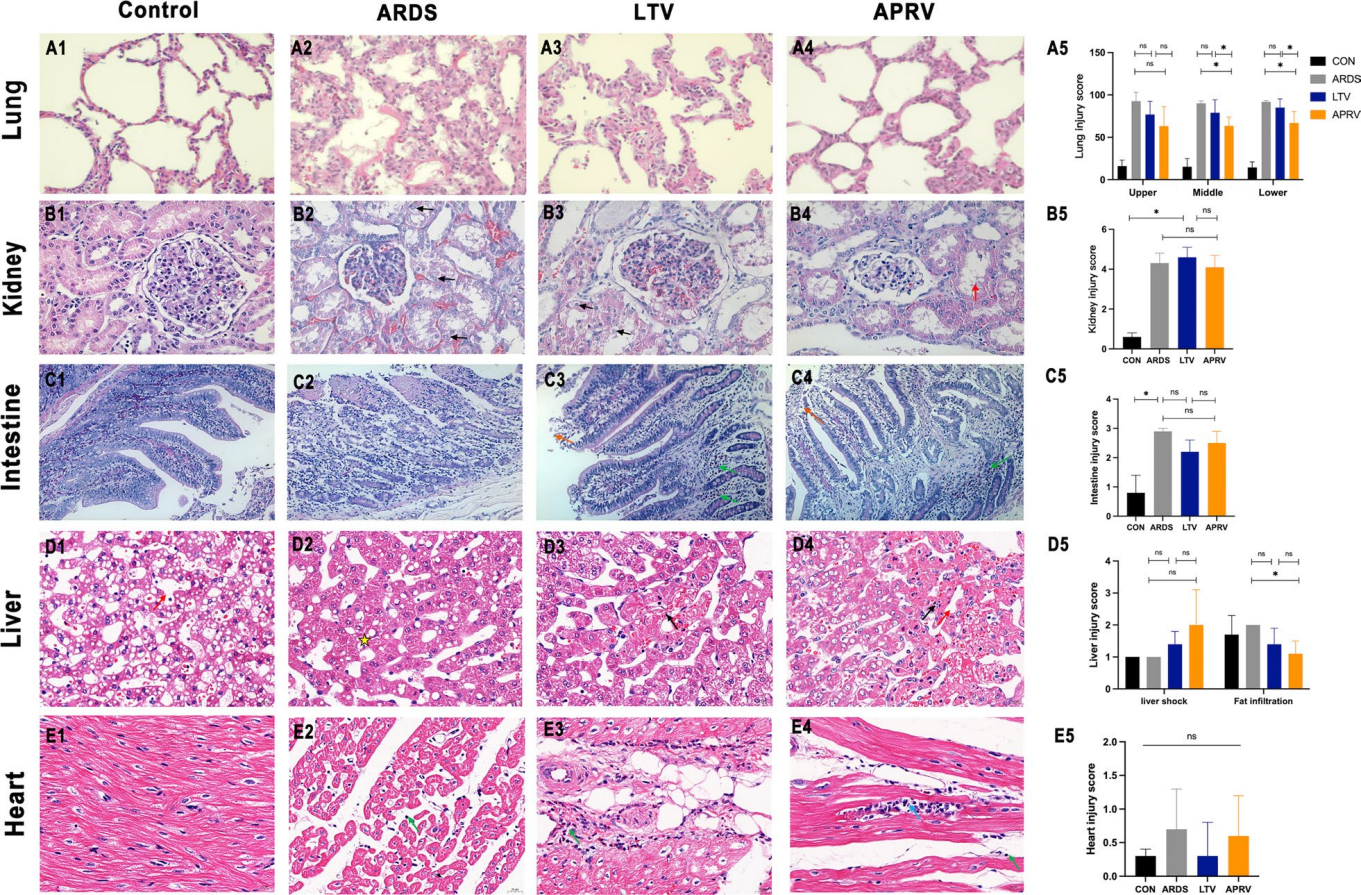
Representative light micrographs of multi-organs in different groups. **A1** lung, **B1** kidney, **C1** intestine, **D1** liver, **E1** heart in the control group; **A2** lung, **B2** kidney, **C2** intestine, **D2** liver, **E2** heart in the ARDS group; **A3** lung, **B3** kidney, **C3** intestine, **D3** liver, **E3** heart in the LTV group; **A4** lung, **B4** kidney, **C4** intestine, **D4** liver, **E4** heart in the APRV group; Histopathologic injury score in lung (**A5**), kidney (**B5**), small intestine (**C5**), liver (**D5**) and heart (**E5**). Black arrow in kidney = renal tubular epithelial cell edema; Red arrow in kidney = epithelial cell damage; Orange arrow in intestine = necrotic detachment of intestinal villous epithelial cells; Black arrow in liver = necrosis; Red arrow in liver = dilated liver sinuses; Yellow star in liver = fat infiltration; Green arrow = inflammatory infiltrates; Blue arrow = fibrous necrosis; small intestine: magnification 200 × ; lung, kidney, liver, heart: magnification 400 × . *p < 0.05 were calculated by Kruskal–Wallis H-test. ns = no significance

Obvious pulmonary hyaline membrane, infiltration of inflammatory cells, protein debris deposition was observed in ARDS, LTV and APRV groups (Fig. 4A1–A4).

Lung injury scores: In the upper lung, the LTV and APRV groups did not have a statistically significant reduction in injury compared to the ARDS group, and there was no statistical significance between the two groups (*p*_upper_ = 0.30). Compared with the ARDS group, the right middle and lower lung injuries were significantly reduced in the APRV group (*p*_middle_ = 0.04, *p*_lower_ = 0.03), while the LTV group had slightly reduction in injury with no statistically significant (*p*_middle_ = 0.34, *p*_lower_ = 0.42). Between the APRV group and the LTV group, there were noticeable differences in the degree of injury to the middle and lower lungs (*p*_middle_ = 0.04, *p*_lower_ = 0.01) (Fig. 4A5).

#### Kidney

In the ARDS, LTV and APRV groups, severe edema, glomerular atrophy and protein mucus exudation were observed (Fig. 4B1–B4).

Paller Scores: The control group had the lowest injury score, compared to the LTV group (*p* = 0.02) and APRV group (*p* = 0.05). The LTV and APRV groups reported similar renal injury followed ARDS, and there was no statistically significant difference between the two groups (*p* = 1.0) (Fig. 4B5).

#### Small intestine

In the ARDS, LTV and APRV groups, the small intestinal glands in the lower mucosal layer were edema, and intestinal villus epithelial cells were necrotic and shed in the interstitium (Fig. 4C1–C4).

Chui’s scores: Compared with the control group, ARDS group (*p* = 0.03), LTV group (*p* = 0.64) and APRV group (*p* = 0.09) had higher degree of histological damage.

Compared with the ARDS group, the LTV group (*p* = 0.84) and APRV group (*p* = 1.0) both had a downward trend with no significant difference. The absence of a significant difference between the two groups indicates that APRV may not cause further harm (*p* = 1.0) (Fig. 4C5).

#### Liver

Liver injury was assessed in two dimensions: hepatic shock and hepatic fatty infiltration. The control and ARDS group did not show significant central lobular congestion and central lobular necrosis, whereas with the use of mechanical ventilation, showed varying degrees of focal hepatocyte necrosis. Small lipid droplets were found in hepatocytes of all four groups (Fig. 4D1–D4).

Liver shock score: Compared with the ARDS group, there was no statistically significant increase in shock scores in the LTV (*p* = 0.21) and APRV groups (*p* = 0.13). Although the mean liver shock score was higher in the APRV group than in the LTV group, there was no statistical difference between the two groups (*p* = 0.14).

Fat infiltration score: Compared with the ARDS group, the LTV and APRV groups had a lower score in fat infiltration (*p* = 0.21, *p* = 0.02). However, there was no statistical difference between the LTV and APRV groups (*p* = 0.13) (Fig. 4D5).

#### Heart

There was no obvious edema, hyperemia, and subendocardial hemorrhage to the hearts of the four groups, with local interstitial inflammatory cells infiltrated in the ARDS, LTV, and APRV groups, mainly lymphocytes. Necrosis was observed in the APRV group (Fig. 4E1–E4).

Heart injury score: There was no statistically significant difference between the four groups. The APRV group had an increased injury score compared to the LTV group, but it was not statistically significant (*p* = 0.20) (Fig. 4E5).

The detailed scores of the pathology scores in multi-organs are shown in the Additional file 2.

### Apoptosis in multi-organ tissues due to mechanical ventilation in ARDS

#### Lung

**Fig. 5:**
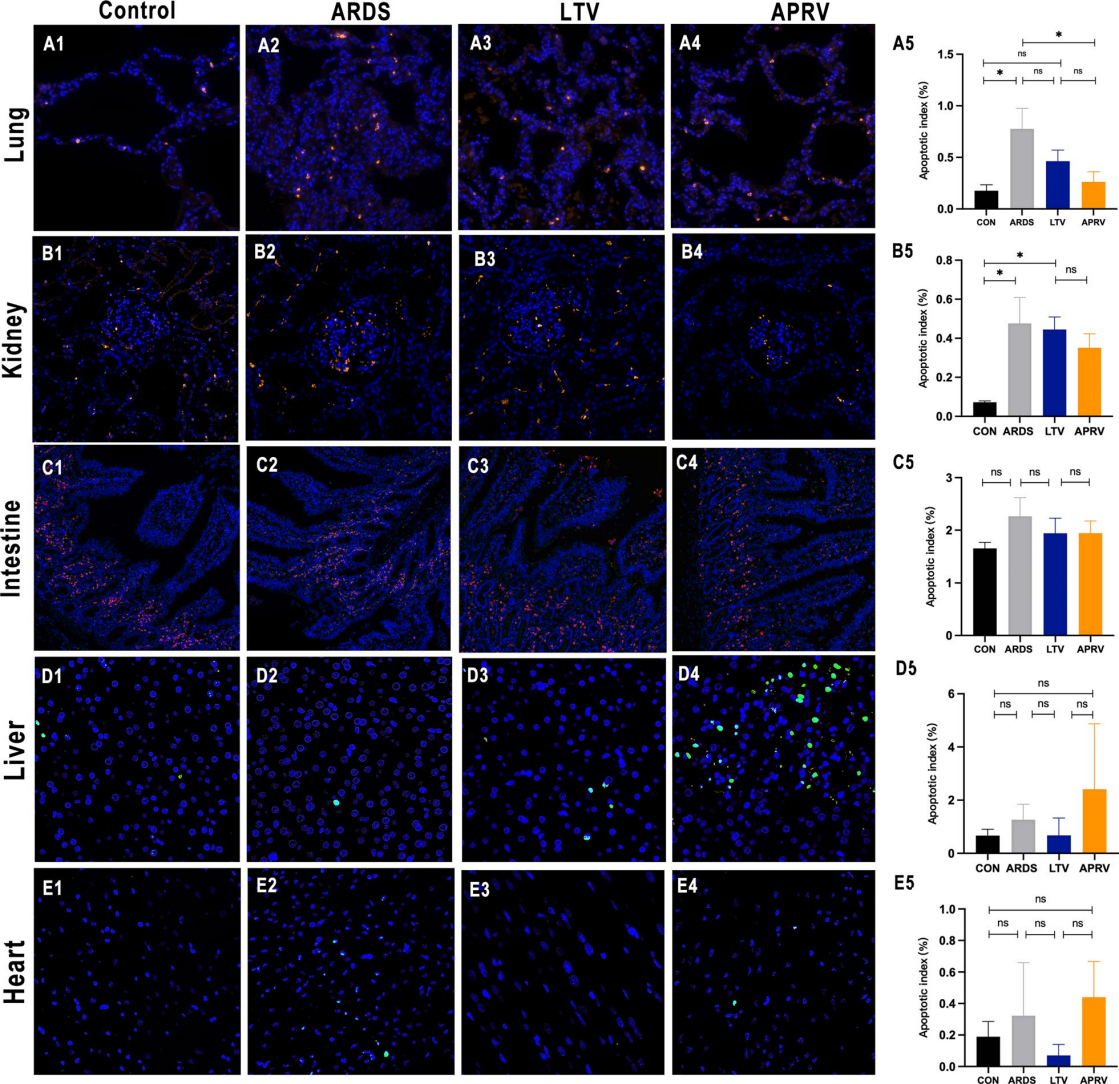
Representative TUNEL staining of multi-organs in different groups. **A1** lung, **B1**kidney, **C1** intestine, **D1** liver, **E1** heart in the control group; **A2** lung, **B2** kidney, **C2** intestine, **D2** liver, **E2** heart in the ARDS group; **A3** lung, **B3** kidney, **C3** intestine, **D3** liver, **E3** heart in the LTV group; **A4** lung, **B4** kidney, **C4** intestine, **D4** liver, **E4** heart in the APRV group; Apoptotic index in lung (**A5**), kidney (**B5**), small intestine (**C5**), liver (**D5**) and heart (**E5**); *p < 0.05; ns = no significance; kidney, small intestine: magnification 200 × ; Lung, liver, heart: magnification 400 × ; Orange in lung and kidney, pink in small intestine, green in liver and heart: TUNEL signal; Blue: DAPI. *p < 0.05 were calculated by Kruskal–Wallis H-test. ns = no significance

The ARDS (*p* = 0.01) showed a increased apoptosis compared to the control group, while APRV groups (*p* = 0.03) reduced the incidence of apoptosis compared to the high rate of apoptosis in the ARDS group. The APRV group had a lower apoptotic index compared to the LTV group, with no statistically significant (*p* = 0.41) (Fig. 5A1–A5).

#### Kidney

The ARDS group showed significant apoptosis compared to the control group (*p* = 0.01), while the LTV (*p* = 1.0) and APRV groups (*p* = 0.87) did not significantly alleviate apoptosis after severe ARDS modeling. There was no statistical difference between the LTV and APRV groups (*p* = 0.70) (Fig. 5B1–B5).

#### Small intestine

Apoptosis of the small intestine was present in the control, ARDS, LTV and APRV groups, but there were no significant differences between the groups (Fig. 5C1–C5).

#### Liver

Although the apoptotic index of hepatocytes tended to increase in the APRV group, there was no statistically significant difference in the apoptotic index compared with the LTV group (*p* = 0.34) (Fig. 5D1–D5).

#### Heart

Increased myocardial tissue apoptosis was observed in the ARDS and APRV groups. There was a tendency for the apoptotic index to increase in the APRV group compared to the LTV group, however, this was not statistically significant (*p* = 0.06) (Fig. 5E1–E5).

### The level of serum inflammatory cytokines

**Fig. 6:**
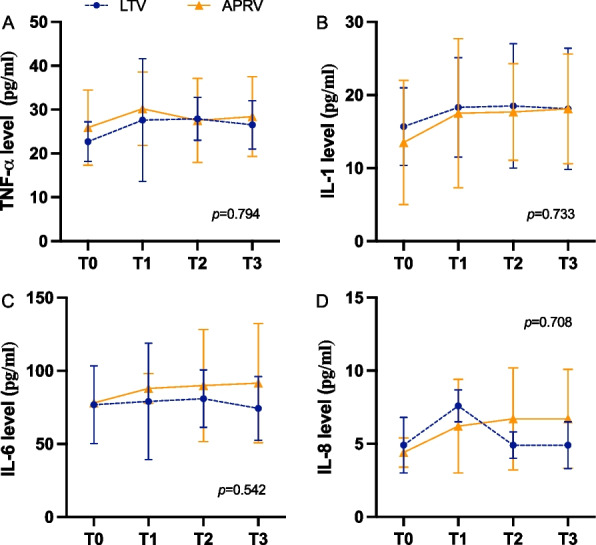
The level of serum inflammatory cytokines. **A** TNF-α level in serum; **B** IL-1 level in serum; **C** IL-6 level in serum; **D** IL-8 level in serum; TNF-α, tumor necrosis factor alpha; IL-1, interleukin 1; IL-6, interleukin 6; IL-8, interleukin 8. The *p*-values were calculated by repeated measurement ANOVA between the two groups

We measured the serum levels of IL-1, IL-6, IL-8, and TNF-α at T0, T1, T2, and T3 in the LTV and APRV groups to further investigate the inflammatory (Fig. 6). Neither the APRV group nor the LTV group exhibited an excessive increase in inflammatory cytokines after ARDS modeling.

## Discussion

APRV, an emerging developed mode of mechanical ventilation, has been shown to offer pulmonary benefits and improve the prognosis of severe ARDS [18, 29, 30]. However, whether the persistent high pressure of APRV will cause damage to extra-pulmonary organs has not been elucidated. In this model of severe ARDS in pigs, APRV improved lung compliance with reduced lung injury, and did not demonstrate more extra-pulmonary organ injuries as compared with LTV.

In consistent with previous findings, the APRV group in this study significantly improved lung compliance in severe ARDS, and although the improvement in oxygenation was not statistically significant compared to the LTV group, a potential advantage could still be seen. The statistical non-significance may be related to the sample size of the groups and the relative short ventilation time. Considering the heterogeneity of ARDS and the gravitational distribution of pulmonary consolidation in severe ARDS [31, 32], we divided the lung tissue into upper, middle and lower for separate lung injury scores. In our study, the lung histological injury score showed that the APRV group significantly reduced the injuries of the middle and lower lobes of lung compared with the LTV group, while the improvement in the upper lungs did not appear to be significant. Our results are consistent with the theory that APRV can fully open the consolidation of the middle lung within a long inspiratory time, allow the gas to be evenly distributed to the more serious parts (lower lung), reduce the repeated shear force between the alveoli with less breathing times, and give the alveoli enough “rest”, which is conducive to the repair of alveolar epithelium.

Before the first organ failure reaches decompensation, most patients in ICU die of complications of the other [6]. The kidney and heart play arguably the most important roles in multi-organ interactions, and their failure is often the terminal of irreversible MODS. Mechanical ventilation in ARDS patients not only affects renal blood flow, stimulating sympathetic nerves to induce renal vasoconstriction and acute kidney injury [33–35], but also lead to increased right ventricular afterload and reduced cardiac output [36]. In the present study, both APRV and LTV increased the pathology score of kidney injury after 48 h ventilation, demonstrating the existence of adverse effects of mechanical ventilation on the kidneys. However, the impairment from mechanical ventilation was not significant in the heart injury score. Kidney and heart injury did not differ significantly between the two modes of mechanical ventilation. Although it has been shown in several studies that maintaining spontaneous breathing in APRV improves renal hemodynamics [37], increases cardiac output and reduces the use of vasoactive drugs [38, 39]. However, in the present study, due to the severity of ARDS modeling and the short observation time, the influence of spontaneous breathing on APRV was slight. Moreover, myocardial injury and apoptosis tended to slightly increase in the APRV group with no statistically significant. This injury may still be related to prolonged intrathoracic pressure, which affects myocardial contraction and relaxation. This finding reminds us that the use of APRV still needs to pay attention to its impact on the heart, and the myocardial pulsation can be observed clinically by ultrasound.

The intestine and liver, as abdominal organs, are also affected by mechanical ventilation. Mechanical ventilation with high positive end expiratory pressure can reduce intestinal blood perfusion and hepatic blood flow, thus exacerbate the progression of MODS in patients with ARDS [40, 41]. In the present study, after 48 h of ventilation, intestinal mucosal injury was significantly increased in the LTV group and APRV group, but the differences between them were not significant, which indicated the APRV group did not show a more pronounced trend of intestine injury than the LTV group. However, both the liver injury score and apoptosis suggested potential liver injury in the APRV group relative to the LTV group. Although previous studies have concluded that maintaining spontaneous breathing was associated not only with better intestinal blood flow but also with better pre-portal organ blood flow, its improvement on hepatic arterial blood flow was not significant [42]. In the APRV group, liver injury may still occur due to inadequate perfusion of the hepatic artery in the absence or faint presence of spontaneous breathing. Therefore, the clinical significance of this study is that patients with severe ARDS in the ICU who have the potential for intra-abdominal organ damage need to be evaluated for the use of APRV and monitored more frequently in clinical use.

Inflammatory cytokines did not change significantly over time with MV after severe ARDS modelling in this experiment, suggesting that protective MV is known to stabilize but not reduce the systemic inflammatory response. Most hypotheses suggest that the effect of mechanical ventilation on extra-pulmonary organs is through inflammatory cytokines circulating in the blood [10, 11]. However, in this study, although MV has an effect on extrapulmonary organs, we speculate that this effect is not caused by inflammatory cytokines but may be caused by hemodynamics. High P_mean_ and long inspiratory time affect intrathoracic pressure, thereby affecting pulmonary circulation and cardiac function. At the same time, high intrathoracic pressure is also transmitted to abdominal pressure, which effects the perfusion of abdominal organs.

There are several limitations in our study. (1) The experimental observation period was still short, and perhaps a longer observation time is needed to observe organ injury obviously, and its functions as well. (2) Recent studies have shown that there are many phenotypes of ARDS. The saline lavage method more closely mimics the direct lung injury and hypo-inflammatory type of ARDS, which does not represent other types induced by indirect lung injury and hyper-inflammatory. Therefore, further validation on a hyper-inflammatory ARDS model induced by infections is required. (3) This large animal experiment can better imitate the patient's condition, but there must be some individual differences in pigs, and many confounding factors in 48 h of mechanical ventilation, which may be related to the pigs' own compensation and decompensation. (4) The sample sizes of the study groups were relatively small, particularly in the control and ARDS groups. (5) The experiment only focuses on histopathological indications of organ injury not the molecular mechanism and function, which should be discussed in the follow-up study. (6) Due to pig-machine asynchrony and short observation time, spontaneous breathing in the APRV group reached 10–20%, which was slightly lower than our target (30%). It may have weakened the benefits of spontaneous breathing in APRV groups.

## Conclusion

In conclusion, in the experimental pig models of severe ARDS induced by repetitive saline lavage, APRV improved lung compliance with reduced lung injury of middle and lower lobes and did not demonstrate more extra-pulmonary organ injuries as compared with LTV.

## Supplementary Information

**Additional file 1.** Supplementary methods of fluid management, ventilator setup adjustment (LTV and APRV group).**Supplementary table 8**. FiO2/PEEP adjustment method in ARDSnet.**Supplementary table 9**. Initiation settings of APRV. **Supplementary table 10**. Titration of APRV.

**Additional file 2.** Supplementary tables of organ injury score. **Supplementary table 1**. Lung injury score (right upper lobe of lung).** Supplementary table 2**. Lung injury score (right middle lobe of lung).** Supplementary table 3**. Lung injury score (right lower lobe of lung).** Supplementary table 4**. Kidney injury score.** Supplementary table 5**. Intestine injury score.** Supplementary table 6**. Liver injury score.** Supplementary table 7**. Heart injury score.

## Data Availability

The data and any material can be shared, please contact the corresponding author Yan Kang (Kangyan@scu.edu.cn).

## Abbreviations

ARDS: Acute respiratory distress syndrome
MODS: Multi-organ dysfunction syndrome
ICU: Intensive care unit
LTV: Low tidal volume ventilation
APRV: Airway pressure release ventilation
MV: Mechanical ventilation
PEEP: Positive end-expiratory pressure
C_stat_: Respiratory system static compliance
PEFR: Peak expiratory flow rate
RR: Respiratory rate
FiO_2_: Fraction of inspired oxygen
V_T_: Tidal volume
P_high_: Pressure during inspiration
P_low_: Pressure during expiration/release phase
P_mean_: Mean airway pressure
T_high_: The time spent at P_high_
T_low_: The time spent at P_low_
H&E: Hematoxylin eosin staining
TNF-α: Tumor necrosis factor alpha
IL-1: Interleukin 1
IL-6: Interleukin 6
IL-8: Interleukin 8
ELISA: Enzyme-linked immunosorbent assay
TUNEL: Terminal deoxynucleotidyl transferase dUTP nick end labeling
PI: Propidium iodide

## References

[1] Meyer NJ, Gattinoni L, Calfee CS (2021). Acute respiratory distress syndrome. Lancet.

[2] Yadav H, Thompson BT, Gajic O (2017). Fifty years of research in ARDS. Is acute respiratory distress syndrome a preventable disease?. Am J Respir Crit Care Med.

[3] Goligher EC, Costa ELV, Yarnell CJ, Brochard LJ, Stewart TE, Tomlinson G, Brower RG, Slutsky AS, Amato MPB (2021). Effect of lowering Vt on mortality in acute respiratory distress syndrome varies with respiratory system elastance. Am J Respir Crit Care Med.

[4] Quílez ME, López-Aguilar J, Blanch L (2012). Organ crosstalk during acute lung injury, acute respiratory distress syndrome, and mechanical ventilation. Curr Opin Crit Care.

[5] Thakur V, Ratho RK, Kumar P, Bhatia SK, Bora I, Mohi GK, Saxena SK, Devi M, Yadav D, Mehariya S (2021). Multi-organ involvement in COVID-19: beyond pulmonary manifestations. J Clin Med.

[6] Khadaroo RG, Marshall JC (2002). ARDS and the multiple organ dysfunction syndrome. Common mechanisms of a common systemic process. Crit Care Clin.

[7] Dorinsky PM, Gadek JE (1989). Mechanisms of multiple nonpulmonary organ failure in ARDS. Chest.

[8] Vieillard-Baron A, Matthay M, Teboul JL, Bein T, Schultz M, Magder S, Marini JJ (2016). Experts' opinion on management of hemodynamics in ARDS patients: focus on the effects of mechanical ventilation. Intensiv Care Med.

[9] Grübler MR, Wigger O, Berger D, Blöchlinger S (2017). Basic concepts of heart-lung interactions during mechanical ventilation. Swiss Med Wkly.

[10] Lang JD, McArdle PJ, O'Reilly PJ, Matalon S (2002). Oxidant-antioxidant balance in acute lung injury. Chest.

[11] Jaecklin T, Otulakowski G, Kavanagh BP (2010). Do soluble mediators cause ventilator-induced lung injury and multi-organ failure?. Intensiv Care Med.

[12] Nosaka N, Martinon D, Moreira D, Crother TR, Arditi M, Shimada K (2020). autophagy protects against developing increased lung permeability and hypoxemia by down regulating inflammasome activity and IL-1β in LPS plus mechanical ventilation-induced acute lung injury. Front Immunol.

[13] Verbrugge SJ, Sorm V, van’t Veen A, Mouton JW, Gommers D, Lachmann B (1998). Lung overinflation without positive end-expiratory pressure promotes bacteremia after experimental Klebsiella pneumoniae inoculation. Intensiv Care Med.

[14] Nahum A, Hoyt J, Schmitz L, Moody J, Shapiro R, Marini JJ (1997). Effect of mechanical ventilation strategy on dissemination of intratracheally instilled *Escherichia coli* in dogs. Crit Care Med.

[15] Sud S, Friedrich JO, Adhikari NKJ, Fan E, Ferguson ND, Guyatt G, Meade MO (2021). Comparative effectiveness of protective ventilation strategies for moderate and severe acute respiratory distress syndrome. A network meta-analysis. Am J Respir Crit Care Med.

[16] Fan E, Del Sorbo L, Goligher EC, Hodgson CL, Munshi L, Walkey AJ, Adhikari NKJ, Amato MBP, Branson R, Brower RG (2017). An official American Thoracic Society/European Society of Intensive Care Medicine/Society of Critical Care Medicine clinical practice guideline: mechanical ventilation in adult patients with acute respiratory distress syndrome. Am J Respir Crit Care Med.

[17] Venkataraman S, Kinsella JP (2018). Airway pressure release ventilation: A therapy in search of a disease?. Am J Respir Crit Care Med.

[18] Zhou Y, Jin X, Lv Y, Wang P, Yang Y, Liang G, Wang B, Kang Y (2017). Early application of airway pressure release ventilation may reduce the duration of mechanical ventilation in acute respiratory distress syndrome. Intensiv Care Med.

[19] Cheng J, Yang J, Ma A, Dong M, Yang J, Wang P, Xue Y, Zhou Y, Kang Y (2022). The effects of airway pressure release ventilation on pulmonary permeability in severe acute respiratory distress syndrome pig models. Front Physiol.

[20] Steimback PW, Oliveira GP, Rzezinski AF, Silva PL, Garcia CS, Rangel G, Morales MM, Lapa ESJR, Capelozzi VL, Pelosi P, Rocco PR (2009). Effects of frequency and inspiratory plateau pressure during recruitment manoeuvres on lung and distal organs in acute lung injury. Intensiv Care Med.

[21] Percie du Sert N, Hurst V, Ahluwalia A, Alam S, Avey MT, Baker M, Browne WJ, Clark A, Cuthill IC, Dirnagl U (2020). The ARRIVE guidelines 2.0: updated guidelines for reporting animal research. PLoS Biol.

[22] Araos J, Alegria L, Garcia A, Cruces P, Soto D, Erranz B, Salomon T, Medina T, Garcia P, Dubó S (2021). Effect of positive end-expiratory pressure on lung injury and haemodynamics during experimental acute respiratory distress syndrome treated with extracorporeal membrane oxygenation and near-apnoeic ventilation. Br J Anaesth.

[23] Matute-Bello G, Downey G, Moore BB, Groshong SD, Matthay MA, Slutsky AS, Kuebler WM (2011). An official American Thoracic Society workshop report: features and measurements of experimental acute lung injury in animals. Am J Respir Cell Mol Biol.

[24] Paller MS, Hoidal JR, Ferris TF (1984). Oxygen free radicals in ischemic acute renal failure in the rat. J Clin Invest.

[25] Chiu CJ, McArdle AH, Brown R, Scott HJ, Gurd FN (1970). Intestinal mucosal lesion in low-flow states. I. A morphological, hemodynamic, and metabolic reappraisal. Arch Surg.

[26] Jeschke MG, Rensing H, Klein D, Schubert T, Mautes AE, Bolder U, Croner RS (2005). Insulin prevents liver damage and preserves liver function in lipopolysaccharide-induced endotoxemic rats. J Hepatol.

[27] Choi JH, Necsoiu C, Wendorff D, Jordan B, Dixon A, Roberts TR, Beely BM, Cancio LC, Batchinsky AI (2019). Effects of adjunct treatments on end-organ damage and histological injury severity in acute respiratory distress syndrome and multiorgan failure caused by smoke inhalation injury and burns. Burns.

[28] Imai Y, Parodo J, Kajikawa O, de Perrot M, Fischer S, Edwards V, Cutz E, Liu M, Keshavjee S, Martin TR (2003). Injurious mechanical ventilation and end-organ epithelial cell apoptosis and organ dysfunction in an experimental model of acute respiratory distress syndrome. JAMA.

[29] Han GJ, Li JQ, Pan CG, Sun JX, Shi ZX, Xu JY, Li MQ (2017). Experimental study of airway pressure release ventilation in the treatment of acute respiratory distress syndrome. Exp Ther Med.

[30] Roy S, Habashi N, Sadowitz B, Andrews P, Ge L, Wang G, Roy P, Ghosh A, Kuhn M, Satalin J (2013). Early airway pressure release ventilation prevents ARDS-a novel preventive approach to lung injury. Shock.

[31] Galvin I, Drummond GB, Nirmalan M (2007). Distribution of blood flow and ventilation in the lung: gravity is not the only factor. Br J Anaesth.

[32] van der Zee P, Gommers D (2019). Recruitment maneuvers and higher PEEP, the so-called open lung concept, in patients with ARDS. Crit Care.

[33] Koyner JL, Murray PT (2008). Mechanical ventilation and lung-kidney interactions. Clin J Am Soc Nephrol.

[34] van den Akker JP, Egal M, Groeneveld AB (2013). Invasive mechanical ventilation as a risk factor for acute kidney injury in the critically ill: a systematic review and meta-analysis. Crit Care.

[35] Darmon M, Clec'h C, Adrie C, Argaud L, Allaouchiche B, Azoulay E, Bouadma L, Garrouste-Orgeas M, Haouache H, Schwebel C (2014). Acute respiratory distress syndrome and risk of AKI among critically ill patients. Clin J Am Soc Nephrol.

[36] Wrigge H, Zinserling J, Hering R, Schwalfenberg N, Stüber F, von Spiegel T, Schroeder S, Hedenstierna G, Putensen C (2001). Cardiorespiratory effects of automatic tube compensation during airway pressure release ventilation in patients with acute lung injury. Anesthesiology.

[37] Hering R, Peters D, Zinserling J, Wrigge H, von Spiegel T, Putensen C (2002). Effects of spontaneous breathing during airway pressure release ventilation on renal perfusion and function in patients with acute lung injury. Intensiv Care Med.

[38] Kaplan LJ, Bailey H, Formosa V (2001). Airway pressure release ventilation increases cardiac performance in patients with acute lung injury/adult respiratory distress syndrome. Crit Care.

[39] Ge H, Lin L, Xu Y, Xu P, Duan K, Pan Q, Ying K (2021). airway pressure release ventilation mode improves circulatory and respiratory function in patients after cardiopulmonary bypass, a randomized trial. Front Physiol.

[40] Putensen C, Wrigge H, Hering R (2006). The effects of mechanical ventilation on the gut and abdomen. Curr Opin Crit Care.

[41] Matuschak GM, Pinsky MR, Rogers RM (1985). Effects of positive end-expiratory pressure on hepatic blood flow and performance. J Appl Physiol.

[42] Hering R, Bolten JC, Kreyer S, Berg A, Wrigge H, Zinserling J, Putensen C (2008). Spontaneous breathing during airway pressure release ventilation in experimental lung injury: effects on hepatic blood flow. Intensiv Care Med.

