# Supplementary material for: Effects of airway pressure release ventilation on multi-organ injuries in severe acute respiratory distress syndrome pig models
Source: BMC Pulm Med. 2022 Dec 7;22:468. doi: 10.1186/s12890-022-02238-x (PMC9730639; doi:10.1186/s12890-022-02238-x)
Supplement: Supplementary file 1 — Additional file 1. Supplementary methods of fluid management, ventilator setup adjustment (LTV and APRV group).Supplementary table 8. FiO2/PEEP adjustment method in ARDSnet.Supplementary table 9. Initiation settings of APRV. Supplementary table 10. Titration of APRV. [file 12890_2022_2238_MOESM1_ESM.docx]

**Supplementary methods**

1. **Protocol**

Female Bama mini swine, weighing 30-35 kg, were fasted for 12 hour but allowed free access to water before the experiments (Laboratory Animal Center, Sichuan University, China). After premedication with intramuscular atropine (0.02-0.05mg/kg), general anesthesia was administered by ear vein injection of sufentanil (0.2μg/kg/h), midazolam (0.2mg/kg/h) and propofol (0.3-1mg/kg/h). Sedation was aimed at no agitation, no respiratory distress, and no hypotension or bradycardia due to deep analgesia.

A continuous intravenous infusion of balanced electrolyte solution or saline (2-4 ml/kg/h) was administered to maintain daily physiological requirements and norepinephrine (0.02-1μg/kg/min) was titrated to maintain a mean arterial pressure (MAP)> 80 mmHg in the pigs for the duration of the experiment. Tracheotomy was performed using a 7.0 mm inner diameter tracheotomy catheter connected to a ventilator (Puritan Bennett™ 840, Medtronic, USA) for baseline settings.

1. **Fluid management**

Fluid overload is an important factor that affects the pulmonary function of ARDS. The general principle of fluid management is to supplement the daily physiological needs, and perform fluid management according to blood pressure and central venous pressure, urine volume, and body fluid loss, mainly to prevent hypotension. Fluid overdose. The whole process of liquid input is controlled by the volume pump, and the medicine is pumped by the micro pump.

**Daily physiological requirements:**

1. The first 5kg: 4 ml/kg/h; the second 5kg: 2 ml/kg/h; the remaining body weight: 0.5ml/kg/h. The daily physiological requirement of a 30 kg experimental pig is about 960 ml, which is in line with the research point of Diehl et al.
2. After anesthesia, the systolic blood pressure of pigs was 138.8±13.3 mmHg, the diastolic blood pressure was 86±9.1mmHg, and the mean arterial pressure (MAP) was 103.2±9.3 mmHg, which was about 10 mmHg higher than that of humans. During the experiment, the MAP was maintained at about 100 mmHg. When the mean arterial pressure is lower than 75 mmHg, first check the depth of analgesia and sedation, and maintain it at a reasonable level. If the central venous pressure is lower than 8 cmH_2_O, increase the fluid pumping volume of 20-60 ml per hour to maintain the central venous pressure at 8-12 cmH_2_O. At this time, if the mean arterial pressure is still lower than 75mmHg, 0.02-1 μg/kg/min of norepinephrine is given and pumped through the central venous micropump. If the blood pressure suddenly drops and the systolic blood pressure is lower than 100mmHg, dopamine 2mg should be injected slowly intravenously, and the dose should be repeated according to the blood pressure. When hypertension occurs, the common clinical causes of hypertension such as pain, hypoxia, and abnormal pipeline should be excluded first, and an appropriate depth of analgesia and sedation should be maintained, and phentolamine should be appropriately used for antihypertensive treatment.
3. During the experiment, blood sodium, potassium, calcium, and glucose concentrations were monitored by blood gas analysis every 4 hours. When it is too high, symptomatic treatment shall be given according to the principles of clinical treatment.

**3. Ventilator setup adjustment**

**The mechanical ventilation goals:**

All animals were initially ventilated with volume assisted-control ventilation (VCV) , according to low tidal volume ventilation, using a Puritan Bennett™ 840 Ventilator (Medtronic, Covidien, United States)，prior to randomization to APRV protocol or low tidal volume ventilation(LTV).

In both groups, the goals of mechanical ventilation were to maintain plateau pressures no more than 30 cm of water, PaO_2_ between 55 and 100 mm Hg or pulse oximeter between 88% and 98%, an arterial pH≥7.30.

**LTV group procedure：**

1. Set initial V_T_ = 6 ml/kg animal weight.
2. Adjust PEEP guided by the FiO_2_/PEEP table (ARDSnet)

**Supplementary table 8 FiO2/PEEP adjustment method in ARDSnet.**

| FiO_2_ | 0.3 | 0.4 | 0.4 | 0.5 | 0.5 | 0.6 | 0.7 | 0.7 | 0.7 | 0.8 | 0.9 | 0.9 | 0.9 | 1.0 |
| --- | --- | --- | --- | --- | --- | --- | --- | --- | --- | --- | --- | --- | --- | --- |
| PEEP | 5 | 5 | 8 | 8 | 10 | 10 | 10 | 12 | 14 | 14 | 14 | 16 | 18 | 18-24 |

If the ratio of PaO_2_: FiO_2_ <100 with FiO_2_ and PEEP combination as above，PEEP levels could be further titrated by the ways of optimum oxygenation or compliance, at the clinician’s discretion. PEEP was increased by steps of 2 cm H_2_O/4min, and PaO_2_ or respiratory system compliance was measured at each step. The optimal PEEP was defined as 2 cm H_2_O below the level of PEEP, where PaO_2_ or compliance dropped more than 10%.

1. Set initial rate to approximate baseline minute ventilation (not > 35 bpm).
2. Adjust V_T_ and RR to achieve plateau pressure, pH goal above.
3. Check P_plat_ (0.5 second inspiratory pause), every 4 hours and after each change in PEEP or V_T_.

If P_plat_ < 30 cmH_2_O, animals still presented severe dyssynchrony: V_T_ may be increased in 1ml/kg animal weight increments to 7 or 8 ml/kg animal weight if P_plat_ remains < 30 cmH_2_O.

If P_plat_ > 30 cmH_2_O: V_T_ should be decreased by 1ml/kg animal weight steps (minimum = 4 ml/kg), with appropriate adjustments in RR to target equivalent minute volume.

1. In the setting of hypotension (mean arterial pressure <60 mmHg), the clinician could further modify PEEP levels, according to animals’ needs；
2. If the animal presented severe respiratory acidosis (pH < 7.15), the respiratory rate was increased to 35 breaths per minute, with titrations made in V_T_ ( Pplat target of 30 cmH_2_o may be exceeded), according to the ARDSnet protocol. If severe respiratory acidosis (pH < 7.15) persisted, NaHCO_3_ was allowed to be given.

**APRV group procedure:**

1. **APRV initiation settings**

**Supplementary table 9 Initiation settings of APRV**

Initiation settings of APRV

1. Under original ventilation with VCV mode, measure P_plat_, R_rs_ and C_stat_ parameters;
2. P_high_：set at the last P_plat_（max 30 cmH_2_O）;
3. P_low_：5cm H_2_O (Use a minimum PEEP level to prevent atelectasis as our usual care);
4. T_low_：
5. First step：Calculation time constants(τ), τ=R(cmH_2_O/L/S)×C( L/cmH_2_O)；
6. Initial T_low_: 1.0τ-1.5τ ;
7. Second step: Titration of T_low_ to more than or equal to 50%
8. PEFR(Peak expiratory flow rate);
9. Third step: Further titration of T_low_ to achieve the angle of expiratory flow deceleration at 45^°^
10. on the flow/time curve, if the tidal volume is accepted;
11. Release frequency:10-14 frequencies per minute;
12. FiO_2_: Same as prior mode, or 100% and reduced to 60% as tolerated;
13. Target spontaneous respiratory level as spontaneous minute ventilation (SV) approximately

30% total minute ventilation (MV_total_).

a. Mild to moderate ARDS: SV equal to 30-60% MVtotal, absent of dyspnea；

b. Severe ARDS：SV equal to 10-30% MVtotal, absent of dyspnea.

Note: VCV volume assisted-control ventilation, P_plat_ plateau airway pressure, R_rs_ respiratory system resistance, C_stat_ static respiratory system compliance, SV spontaneous minute ventilation.

**B. APRV Titration**

**Supplementary table 10 Titration of APRV**

**Titration of APRV**

**Hypercapnia**

1. Ensure animal is not over sedated and achieve the SV level.
2. Increase release volume：

➀. Increase △P (P_high_-P_low_):

a. Increase P_high_ by 1- 2cmH_2_O per increment

(max 30cmH_2_O);

b. Decrease P_low_ by 1-2cmH_2_O as necessary;

➁. Prolong T_low_ 0.05-0.1s, to 50% of PEFR.

1. Improve release minute ventilation by increasing release frequency:

Increase release frequency by 1-2 rate per increment

(Max 30 frequencies/minute)

**Severe hypercapnia (PH≤7.2, PaCO_2_>60mmHg)**

Increase △P simultaneously increase release frequency

1. If hypercapnia is not responsive to all the above adjustments,

APRV was transmitted to other treatment.

1. Increase P_high_ by 1-2cmH_2_O per increment (max 30cmH_2_O);
2. Without CO_2_ retention, prolong Thigh:

➀ Decrease T_low_ 0.05-0.1s, (minimal τ);

➁ Increase P_low_ by 1-2cmH_2_O as necessary;

➂Decrease release frequency by 1-2 rate per decrement;

1. Combine recruitment maneuver or prone potion;
2. With severe CO_2_ retention, improve minute ventilation;
3. Ensure the sedation and SV target level;
4. Increase FiO_2_;
5. If hypoxemia is not responsive to all the above adjustments, APRV was transmitted to other treatment.

**Hypoxemia**

**According to respiratory mechanics, ventilation parameters, arterial blood gas analysis, hemodynamic parameters.**

Note: SV denotes spontaneous breath minute ventilation. PEFR peak expiratory flow rate, P_high_, P_low_, T_high_, T_low_ and FiO_2_ were titrated based on interpretation of the measured respiratory mechanics, such as expiratory flow waveform, respiratory system compliance, airway resistance, release volumes (also defined as monitoring tidal volumes), MV_total_, SV, PEEP, P_peak_, P_plat_, and arterial blood gases, and bedside lung ultrasound.
