# Supplementary material for: Effects of airway pressure release ventilation on multi-organ injuries in severe acute respiratory distress syndrome pig models
Source: BMC Pulm Med. 2022 Dec 7;22:468. doi: 10.1186/s12890-022-02238-x (PMC9730639; doi:10.1186/s12890-022-02238-x)
Supplement: Supplementary file 2 — Additional file 2. Supplementary tables of organ injury score. Supplementary table 1. Lung injury score (right upper lobe of lung). Supplementary table 2. Lung injury score (right middle lobe of lung). Supplementary table 3. Lung injury score (right lower lobe of lung). Supplementary table 4. Kidney injury score. Supplementary table 5. Intestine injury score. Supplementary table 6. Liver injury score. Supplementary table 7. Heart injury score. [file 12890_2022_2238_MOESM2_ESM.docx]

**Supplementary tables**

**Supplementary table 1 Lung injury score(right upper lobe of lung).**

| Upper lobe | CON | ARDS | LTV | APRV |
| --- | --- | --- | --- | --- |
| Neutrophils in the alveolar space | 0.4(0.3)^a^ | 1.9(0.2) | 1.4(0.5) | 1.0(0.6) |
| Neutrophils in the interstitial space | 0.5(0.1) | 1.9(0.2) | 1.9(0.1) | 1.6(0.6) |
| Hyaline membranes | 0.0(0.0)^b^ | 0.6(0.3) | 1.3(0.5) | 0.9(0.5) |
| Proteinaceous debris filling the airspaces | 0.1(0.1)^ab^ | 1.9(0.1) | 1.6(0.3) | 1.3(0.5) |
| Alveolar septal thickening | 0.2(0.3)^b^ | 0.9(0.3) | 1.0(0.3) | 0.7(0.3) |
| Total | 16.0(7.0)^ab^ | 92.7(10.3) | 76.9(15.5) | 63.3(22.9) |

^a^*p*<0.05 compared to the ARDS group at this time point in Kruskal-Wallis H-test.

^b^*p*<0.05 compared to the LTV group at this time point in Kruskal-Wallis H-test.

^c^*p*<0.05 compared to the APRV group at this time point in Kruskal-Wallis H-test.

**Supplementary table 2 Lung injury score(right middle lobe of lung).**

| Middle lobe | CON | ARDS | LTV | APRV |
| --- | --- | --- | --- | --- |
| Neutrophils in the alveolar space | 0.3(0.3)^ab^ | 2.0(0.0) | 1.5(0.5) | 1.2(0.3)^a^ |
| Neutrophils in the interstitial space | 0.5(0.4)^ab^ | 1.9(0.2) | 1.9(0.3) | 1.6(0.3) |
| Hyaline membranes | 0.0(0.0)^abc^ | 1.0(0.3) | 1.2(0.5) | 0.9(0.4) |
| Proteinaceous debris filling the airspaces | 0.2(0.2)^ab^ | 2.0(0.0) | 1.8(0.2) | 1.4(0.2)*^a^ |
| Alveolar septal thickening | 0.1(0.2) | 1.2(0.3) | 1.2(0.6) | 0.6(0.6) |
| Total | 15.3(9.5)^ab^ | 90.2(2.8) | 79.0(15.3) | 63.6(10.3)*^a^ |

^a^*p*<0.05 compared to the ARDS group at this time point in Kruskal-Wallis H-test.

^b^*p*<0.05 compared to the LTV group at this time point in Kruskal-Wallis H-test.

^c^*p*<0.05 compared to the APRV group at this time point in Kruskal-Wallis H-test.

*p<0.05 compared to the LTV group at this time point in Kruskal-Wallis H-test.

**Supplementary table 3 Lung injury score(right lower lobe of lung).**

| Lower lobe | CON | ARDS | LTV | APRV |
| --- | --- | --- | --- | --- |
| Neutrophils in the alveolar space | 0.5(0.5)^ab^ | 2.0(0.0) | 1.7(0.3) | 1.4(0.4)^a^ |
| Neutrophils in the interstitial space | 0.2(0.2)^ab^ | 2.0(0.0) | 2.0(0.1) | 1.5(0.4)*^a^ |
| Hyaline membranes | 0.0(0.1)^ab^ | 1.1(0.2) | 1.1(0.6) | 0.7(0.4) |
| Proteinaceous debris filling the airspaces | 0.1(0.2)^ab^ | 2.0(0.0) | 1.8(0.3) | 1.5(0.3)*^a^ |
| Alveolar septal thickening | 0.1(0.2)^ab^ | 1.0(0.2) | 1.3(0.4) | 0.6(0.4)* |
| Total | 14.6(6.5)^ab^ | 91.9(1.5) | 85.1(10.2) | 66.9(13.6)*^a^ |

^a^*p*<0.05 compared to the ARDS group at this time point in Kruskal-Wallis H-test.

^b^*p*<0.05 compared to the LTV group at this time point in Kruskal-Wallis H-test.

^c^*p*<0.05 compared to the APRV group at this time point in Kruskal-Wallis H-test.

*p<0.05 compared to the LTV group at this time point in Kruskal-Wallis H-test.

**Supplementary table 4 Kidney injury score.**

|  | CON | ARDS | LTV | APRV |
| --- | --- | --- | --- | --- |
| Renal tubule dilatation | 0.1(0.1) | 0.4(0.5) | 0.6(0.4) | 0.6(0.3) |
| Tube type | 0.0(0.0) | 0.1(0.2) | 0.3(0.3) | 0.1(0.2) |
| Cell debris | 0.1(0.1)^abc^ | 1.0(0.0) | 1.0(0.0) | 0.9(0.2) |
| Granular degeneration of epithelial cells | 0.0(0.0)^abc^ | 1.0(0.0) | 0.9(0.1) | 0.8(0.2) |
| Vacuolar degeneration | 0.4(0.2)^abc^ | 0.9(0.2) | 1.0(0.0) | 1.0(0.1) |
| Nuclear pyknosis | 0.0(0.0)^abc^ | 0.9(0.1) | 0.8(0.2) | 0.7(0.2) |
| total | 0.6(0.2)^b^ | 4.3(0.5) | 4.6(0.5) | 4.1(0.6) |

^a^*p*<0.05 compared to the ARDS group at this time point in Kruskal-Wallis H-test.

^b^*p*<0.05 compared to the LTV group at this time point in Kruskal-Wallis H-test.

^c^*p*<0.05 compared to the APRV group at this time point in Kruskal-Wallis H-test.

**Supplementary table 5 Intestine injury score.**

|  | CON | ARDS | LTV | APRV |
| --- | --- | --- | --- | --- |
| Total | 0.8(0.6)^ac^ | 2.9(0.1) | 2.2(0.4) | 2.5(0.4) |

^a^*p*<0.05 compared to the ARDS group at this time point in Kruskal-Wallis H-test.

^b^*p*<0.05 compared to the LTV group at this time point in Kruskal-Wallis H-test.

^c^*p*<0.05 compared to the APRV group at this time point in Kruskal-Wallis H-test.

**Supplementary table 6 Liver injury score.**

|  | CON | ARDS | LTV | APRV |
| --- | --- | --- | --- | --- |
| liver shock | 1.0(0.0) | 1.0(0.0) | 1.4(0.4) | 2.0(1.1) |
| Fat infiltration | 1.7(0.6) | 2.0(0.0) | 1.4(0.5) | 1.1(0.4)^a^ |

^a^*p*<0.05 compared to the ARDS group at this time point in Kruskal-Wallis H-test.

**Supplementary table 7 Heart injury score.**

|  | CON | ARDS | LTV | APRV |
| --- | --- | --- | --- | --- |
| Edema | 0(0) | 0(0) | 0(0) | 0(0) |
| Degeneration | 0(0) | 0(0) | 0(0) | 0.075(0.14) |
| Inflammation | 0.3(0.1) | 0.7(0.6) | 0.3(0.5) | 0.5(0.4) |
| Hyperemia | 0(0) | 0(0) | 0(0) | 0(0) |
| Subendocardial hemorrhage | 0(0) | 0(0) | 0(0) | 0(0) |
| Total | 0.3(0.1) | 0.7(0.6) | 0.3(0.5) | 0.6(0.6) |
